# High-Consistency Optical Fiber Fabry–Perot Pressure Sensor Based on Silicon MEMS Technology for High Temperature Environment

**DOI:** 10.3390/mi12060623

**Published:** 2021-05-28

**Authors:** Fei Feng, Pinggang Jia, Jiang Qian, Zhengpeng Hu, Guowen An, Li Qin

**Affiliations:** Science and Technology on Electronic Test and Measurement Laboratory, North University of China, Taiyuan 030051, China; fei3188002@163.com (F.F.); pgjia@nuc.edu.cn (P.J.); QJiangnuc@163.com (J.Q.); zhengpengcn@163.com (Z.H.); anguowen@nuc.edu.cn (G.A.)

**Keywords:** MEMS, Fabry–Perot, fiber optic sensors, pressure measurement, high-temperature

## Abstract

This paper proposes a high-temperature optical fiber Fabry–Perot pressure sensor based on the micro-electro-mechanical system (MEMS). The sensing structure of the sensor is composed of Pyrex glass wafer and silicon wafer manufactured by mass micromachining through anodic bonding process. The separated sensing head and the gold-plated fiber are welded together by a carbon dioxide laser to form a fiber-optic Fabry–Perot high temperature pressure sensor, which uses a four-layer bonding technology to improve the sealing performance of the Fabry–Perot cavity. The test system of high temperature pressure sensor is set up, and the experimental data obtained are calculated and analyzed. The experimental results showed that the maximum linearity of the optical fiber pressure sensor was 1% in the temperature range of 20–400 °C. The pressure sensor exhibited a high linear sensitivity of about 1.38 nm/KPa at room temperature at a range of pressures from approximarely 0-to 1 MPa. The structure of the sensor is characterized by high consistency, which makes the structure more compact and the manufacturing process more controllable.

## 1. Introduction

High-temperature pressure sensors have been widely used in the aerospace industry and in turbine engines, oil wells, reactors, and steam turbines [1,2,3,4,5,6]. In recent decades, various high-temperature and high-pressure sensors have been developed based on different working principles [7,8]. Currently, silicon fiber Fabry–Perot (FP) pressure sensors, along with their advantages, have attracted the interest of scientists nationally and internationally. Liao et al. developed a FP fiber probe based on submicron silica, which is an improved arc discharge sensor technology [9]. Li et al. developed a new type of micro-FP sensor, which made from an optical fiber welding machine to measure intracranial pressure [10]. Zhang et al. proposed a femto-second laser micromachining method for a diaphragm-type Fabry–Perot fiber sensor to measure pressures at high temperature [11]. Zhu et al. proposed a miniature high-temperature optical fiber pressure sensor that was customized at the head end of a single-mode optical fiber by fusion, cutting, and wet chemical etching [12]. Wang et al. proposed a Fabry–Perot fiber interferometer and a pressure and temperature multiplex sensor system based on a fiber Bragg grating [13], which is helpful for the permanent detection of the downhole pressure and temperature in high- temperature oil wells.

Recently, some FP fiber sensors based on micro-electro-mechanical systems (MEMS) technology have been developed and used, e.g., to measure ultrasound, pressure, and acceleration [14,15,16,17,18]. Yin et al. proposed a Fabry–Perot structure sensor based on a combination of pressure and temperature; this sensor can also be mass produced [19]. Their proposed silicon-based sensor head chip is connected to a single-mode optical fiber using a UV-curable epoxy resin. Hill et al. proposed a FP fiber pressure sensor with a cavity length of 300 μm, the structure of which uses an SU-8 diaphragm as the reflection film of the FP interferometer. Zhu et al. developed an FP pressure sensor, the structure of which is composed of SU-8 glue and an angled optical fiber [20]. The single-mode angled fiber is pressed into the groove formed on the silicon surface with an SU-8 photo-resist. Pang et al. developed a Fabry–Perot sensor based on MEMS for the simultaneous measurement of pressure and temperature [21]. The structure of the sensor enables the user to select a high-temperature polymer epoxy resin-sealed cavity and to affix the optical fiber to the sensor. In the above-mentioned MEMS Fabry–Perot optical fiber sensor, the sensor head and the optical fiber are glued together, which makes the complete sealing of the sensor impossible at high temperatures, and causes a relatively large temperature drift.

In this paper, an optical fiber Fabry–Perot pressure sensor based on MEMS and CO_2_ laser fusion technology was developed and verified by experiments in a high-temperature environment. The sensing head of the sensor was batch manufactured by anodic bonding. The sensor head involved four-layer anodic bonding of a Pyrex glass sheet and a partially gold-plated silicon wafer. In order to prevent thermal matching, a gold-plated optical fiber was used for laser fusion with the sensor head. On this basis, the demodulation system and experimental device of the optical fiber Fabry–Perot pressure sensor were established, and the sensing characteristics of the sensor in the temperature range of 20–400 °C were tested and analyzed.

## 2. Principle and Simulation of Sensor

### 2.1. Working Principle

**Figure 1:**
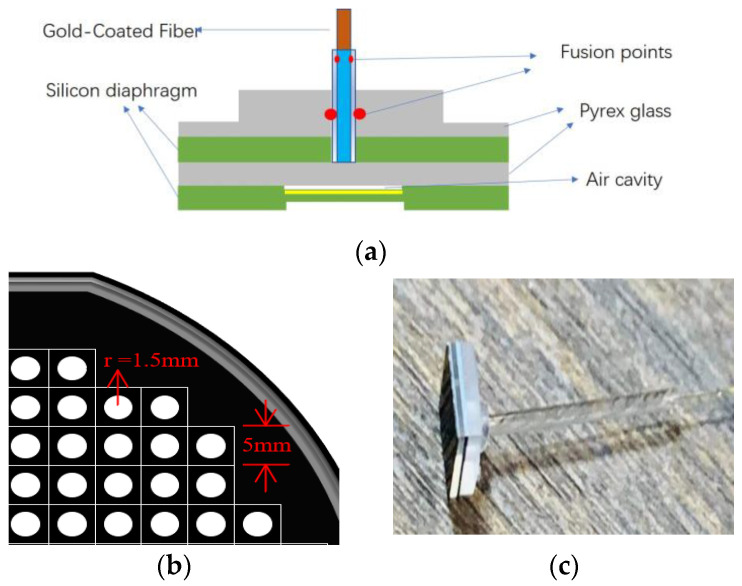
(**a**) The sensor schematic; (**b**) the etched mask structure; (**c**) the side view of the sensor.

Figure 1 shows the physical and structural diagram of the FP optical fiber pressure sensor, which mainly comprised a gold-coated fiber (GCF), glass tube, Pyrex glass substrate, and silicon diaphragm. The schematic diagram of the sensor is shown in Figure 1a. The sensor head consisted of a micro-machined Pyrex glass wafer, silicon wafer, Pyrex glass wafer, and local gold-plated silicon wafer, affixed using anodic bonding technology. The FP cavity of the sensor was formed by fusing the GCF end face with the glass wafer using a CO_2_ laser. In order to improve the contrast of the interference fringes, a layer of gold film was plated on the inner surface of the silicon on the reflection surface of the FP cavity. The mask structure etched by the sensor is shown in Figure 1b. The side view of the sensor is shown in Figure 1c.

**Figure 2:**
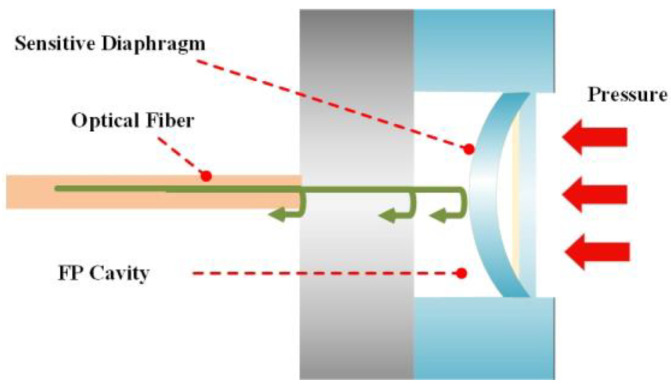
Schematic diagram of diaphragm deformation under vertical uniform pressure.

When pressure is applied to the FP optical fiber pressure sensor, the deformation of the silicon film caused by the measured pressure is directly converted into the length change in the FP cavity, as shown in Figure 2. The change in pressure can be calculated by measuring the length of the FP cavity.

The principle of the sensitivity of the diaphragm is based on the theory of small deflection deformation in elastic mechanics, which means that the deflection of the sensitive silicon diaphragm under a load is miniscule compared to the plate thickness h (w << h). According to the theory of small deflection, the deformation of the center position of the sensitive silicon diaphragm can be calculated by the following equation:

(1) $$\frac{{Pr}^{4}}{Eh^{4}}=\frac{16}{3\left(1-u^{2}\right)}\frac{y}{h}$$

where *P*, *r*, *E*, *h*, *u*, and *y* are the pressure on the diaphragm, the radius of the effective pressure position of the diaphragm, the Young’s modulus of the circular diaphragm material, the thickness of the circular diaphragm, the Poisson’s ratio of the shaped diaphragm material, and the deformation of the diaphragm after compression, respectively. According to elasticity theory, the center deflection of the circular sensor diaphragm is Equation (2),

(2) $$y=\frac{3\left(1-u^{2}\right)P}{16Eh^{3}}r^{4}$$

According to Equation (2), if the radius and thickness of the diaphragm can be determined, and the influence of temperature on the Poisson ratio, radius, and thickness of the diaphragm can be ignored, this indicates that the sensitivity is inversely proportional to Young’s modulus. As the Young’s modulus of silicon film is a function of temperature, the sensitivity is closely related to the temperature. The larger radius and smaller thickness of the silicon diaphragm can provide a higher-pressure sensitivity. Therefore, we can obtain the required pressure sensitivity and pressure measurement range by flexibly designing the radius and thickness of the silicon diaphragm.

According to Figure 1, when light enters the optical fiber, the light is a supercontinuum source, at first, partially reflected. Next, the transmitted light is reflected multiple times between the end face of the glass wafer and the end face of the silicon gold coating. The light is then reflected back to the glass wafer multiple times, leading to multi-beam interference. According to the principle of multi-beam interference, the interference spectrum *I* is determined by:

(3) $$I=I_{1}+I_{2}-2\sqrt{I_{1}I_{2}}\cos(\phi ),$$

where *I*_1_ and *I*_2_ are the intensities of incident light and transmitted light, respectively.

The frequency spectrum of the pressure signal is due to the interference of light in the FP cavity. When the pressure changes, the gold-plated silicon film deforms, which, in turn, changes the length of the FP cavity. The spectrum of the pressure changes the pressure signal. In addition, the pressure signal is demodulated by the demodulator, and the sensor then obtains the position of the light signal.

### 2.2. Design of Diaphragm Structure Parameters

The metal composite film coated on the inner side of the sensitive silicon diaphragm and the glass end surface constitute the two reflective surfaces of the extrinsic FP cavity. When the sensitive silicon diaphragm is subjected to external pressure, the diaphragm is deformed, causing a change in the cavity length of the FP cavity, which shifts the interference spectrum of the sensor. Through the relationship between the movement of the interference spectrum and the length of the Fabry–Perot cavity, the external pressure can be demodulated.

According to the design principle of the sensitive silicon diaphragm and the elastic mechanics theories of small deflection and deformation, MATLAB software was used to simulate the thickness (h) of the sensitive silicon diaphragm and the effective radius (r) of the sensitive diaphragm. The sensitive silicon diaphragm adopts a single-crystal silicon form with a crystal orientation of <100>, the parameters of which (at room temperature) are shown in Table 1.

**Figure 3:**
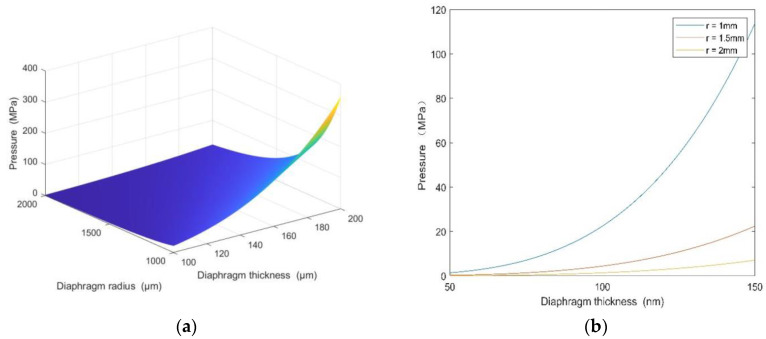
(**a**) The relationships between the pressure range of the sensor, the diameter of the diaphragm, and the thickness of the diaphragm. (**b**) The relationship between the pressure range of the sensor and the thickness of the diaphragm when the radii are 1, 1.5, and 2 mm.

According to Figure 3, the smaller the sensitive diaphragm radius (r) and the larger the diaphragm thickness (h), the larger the maximum pressure measurement range of the sensor.

**Figure 4:**
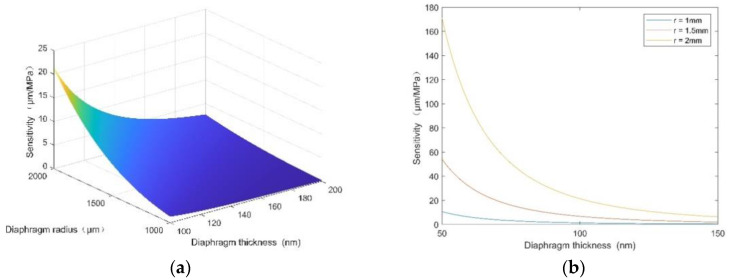
(**a**)The relationship between the sensor sensitivity, diaphragm radius, and diaphragm thickness. (**b**) The relationship between the sensor sensitivity and diaphragm thickness when the radii are 1, 1.5, and 2 mm.

According to Figure 4, the larger the sensitive diaphragm radius (r) and the smaller the diaphragm thickness (h), the greater the sensitivity of the sensor. At the same time, the stress of the sensitive diaphragm is also very high. In order to ensure the normal and safe use of the sensitive diaphragm, its maximum stress should be less than 20% of the maximum breaking stress of the material. This is because the maximum stress generated by the diaphragm would restrict the sensitivity of the sensor and then affect the range of the sensor. In order to optimize the sensitivity and range of the sensor, the diameter and thickness of the silicon diaphragm were set to 3 mm and 186 μm, respectively.

## 3. Manufacturing and Design of Sensors

This section provides a concise and precise description of the experimental results and their interpretation, as well as the experimental conclusions that can be drawn.The pressure-sensitive diaphragm in the manufacturing process of the FP optical fiber pressure sensor using MEMS technology consists of a 4-inch (10.16 cm) silicon wafer with a thickness of approximately 200 μm, which is polished on both sides. The thicknesses of the Pyrex 7740# glass wafer substrate, the silicon diaphragm between the Pyrex7740# glass wafers, and the middle glass wafer of the Pyrex7740# glass wafer are approximately 2 mm, 300 μm, and 500 μm, respectively.

**Figure 5:**
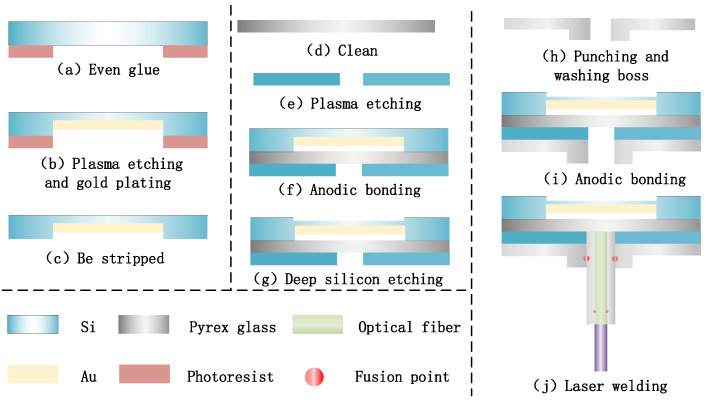
Process development of optical fiber FP pressure sensor.

The manufacturing process of the FP optical fiber pressure sensor is mainly divided into four steps. In the first step, as shown in Figure 5a–c, with the help of a pre-masking process, the bottom surface of the silicon wafer is coated with a photoresist layer of about 10 μm and deep silicon etching 15 μm, and then gold film is sprayed onto the inner surface of the battery using magnetron sputtering deposition technology to increase its reflectivity. After that, the photoresist layer is rinsed off in an acetone solution, and the round gold film is left in the center. The second step is shown in Figure 5d–g, where another photoresist layer is applied. Then, dry etching is used to reduce the thickness of the center of the diaphragm to increase the sensitivity, before the photoresist is dissolved. The third step is shown in Figure 5h–i, where holes are mechanically drilled into the Pyrex glass substrate with a diameter of about 1.0 mm and a groove with a depth of 500 μm; then, the silicon wafer is anodically bonded to the Pyrex glass wafer. The boss structure is then processed on the back of the Pyrex glass, with an outer diameter of 2 mm and a height of 1 mm. Using this method, a batch of wafer-level FP optical fiber pressure sensors are successfully prepared. Finally, in step 4, shown in Figure 5j, the wafer is divided into small pieces with a diameter of 5 mm. Then, a GCF is inserted into a Pyrex tube with an inner diameter and an outer diameter of approximately 126 μm and 1.0 mm, respectively. The optical fiber is placed in a Pyrex tube to protect the structure and to avoid pressure squeezing. The glass tube is inserted into the through hole, and then a CO2 laser is used to fuse it with the substrate. Compared with glue-sealing processes using epoxy resin and inorganic cement, the FP optical fiber pressure sensor welded using a CO2 laser can eliminate the mismatch between various materials at high temperatures, and can adapt better to harsh environments.

## 4. Experimental Results

**Figure 6:**
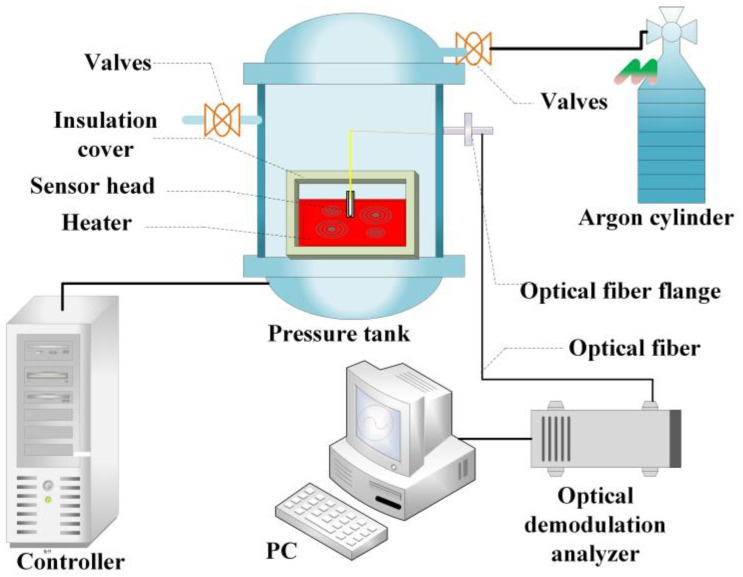
High-temperature test experimental device for the FP optical fiber pressure sensor.

As shown in Figure 6, an experimental device measuring the characteristics of the FP optical fiber pressure sensor in a high-temperature environment was established, including a testing system for the FP optical fiber pressure sensor, a pressure control system, and a temperature control system. Among them, the FP optical fiber pressure sensor sensing system consists of an FP optical fiber pressure sensor, an optical demodulator, an optical fiber flange, and a computer. The FP optical fiber pressure sensor is connected to the optical demodulator using a sealed optical fiber connector. The pressure control system consists of a pressure tank, an argon cylinder, a calibration pressure sensor, and a digital pressure indicator. The pressure in the storage tank is automatically controlled by the pressure control system, and a constant-pressure environment is created by filling the tank with argon gas. A pressure reference is provided for the calibrated pressure sensor and pressure indicator. The pressure control accuracy of the experimental system is 0.1 MPa. The temperature control system consists of a heater, a tank insulation device, a temperature controller, a calibrated thermocouple temperature sensor, and a digital temperature indicator. The temperature control accuracy of the experimental system is 0.1% of the full scale. The heater is placed in a pressure tank to create a high-temperature environment. The insulated area consists of a porous and gas-permeable material to ensure that the pressures inside and outside the area are equal. The calibrated thermocouple temperature sensor and temperature indicator provide a temperature reference.

When the experiment was carried out, the fabricated FP optical fiber pressure sensor was placed in a high-temperature pressure tank near the thermocouple temperature sensor and heater. The initial cavity length of the FP optical fiber pressure sensor was 14,301 nm. At a room temperature of about 20 °C, the pressure in the tank ranged from approximately 0 to 1 MPa (relative pressure), with a step of 100 KPa, and the pressure tank was boosted. Each pressure value was maintained for 5 min while the length of the corresponding cavity was recorded. Next, the temperature in the holding area gradually increased to 400 °C. Similarly, each temperature value was maintained for 5 min while the length of the corresponding cavity was recorded. During the heating process, the temperatures were 20 °C, 100 °C, 200 °C, 300 °C, and 400 °C.

**Figure 7:**
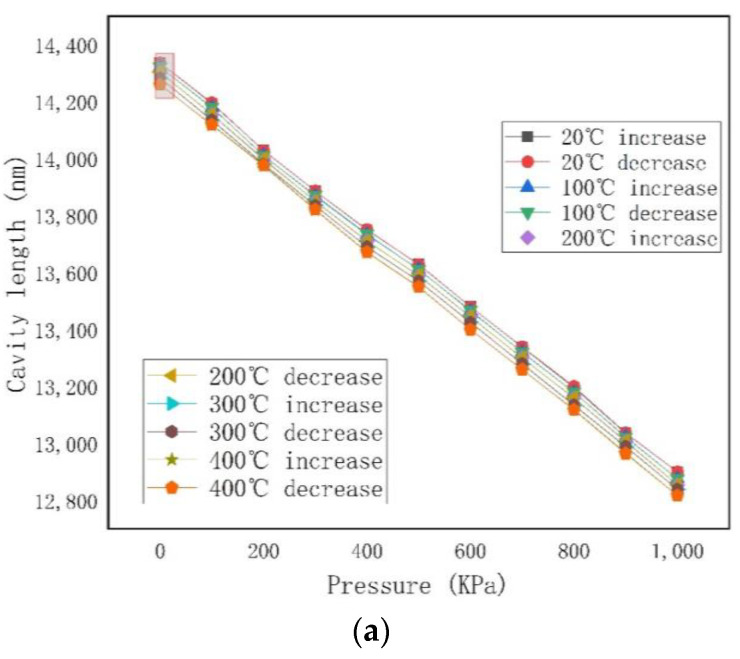

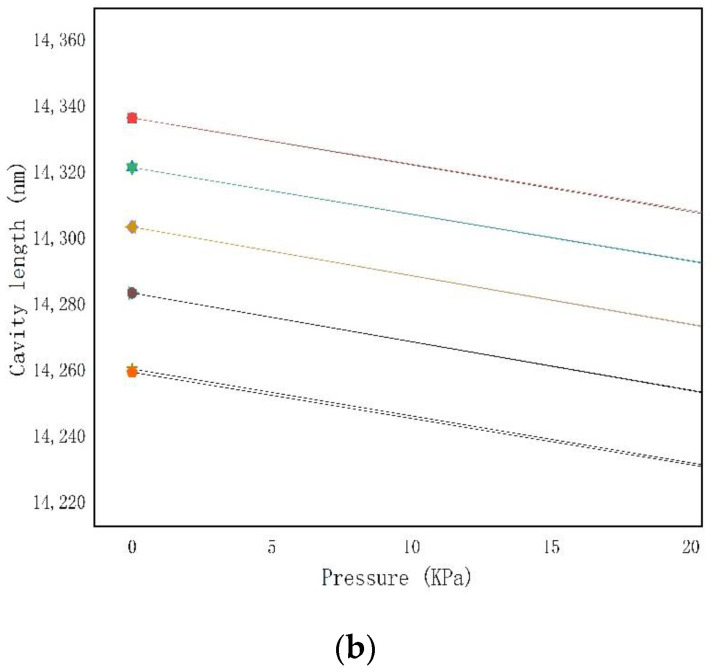
(**a**) The relationship between cavity length and pressure at 20, 100, 200, 300 and 400 °C. (**b**) Partial enlarged drawing.

Figure 7a shows the relationship between the cavity length and pressure between 20 °C and 400 °C. The results show that the length of the Fabry–Perot cavity of the FP optical fiber pressure sensor gradually decreased with the pressure and temperature, respectively. This was due to the gold plating of the reflective surface, which suppressed the cavity length expansion of the sensor. The maximum nonlinear range of the sensor was 0.4% at different working temperatures. At 20 °C, 100 °C, 200 °C, 300 °C, and 400 °C, the sensitivities of the sensor were 1.37924, 1.38009, 1.38151, and 1.38275, respectively, with pressure changes of 1.38395 nm/KPa. This was due to the gold plating on the reflector, which restrained the cavity expansion of the sensor. Figure 7b is a partial enlarged view.

**Figure 8:**
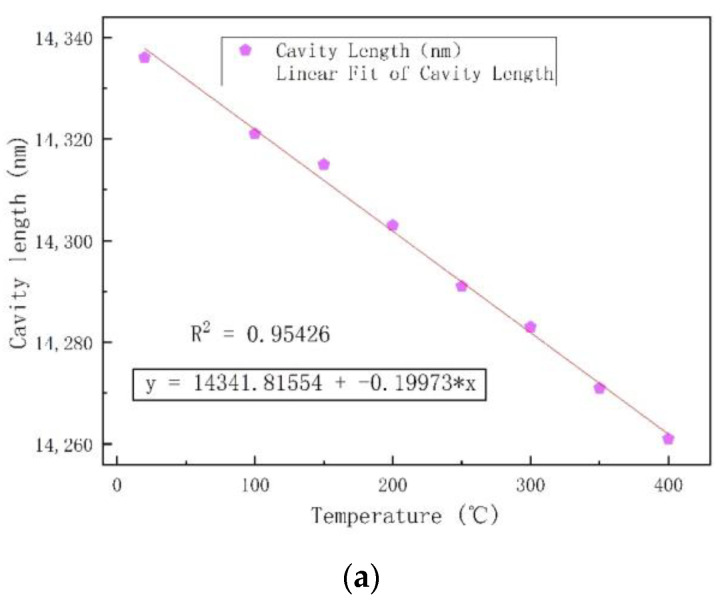

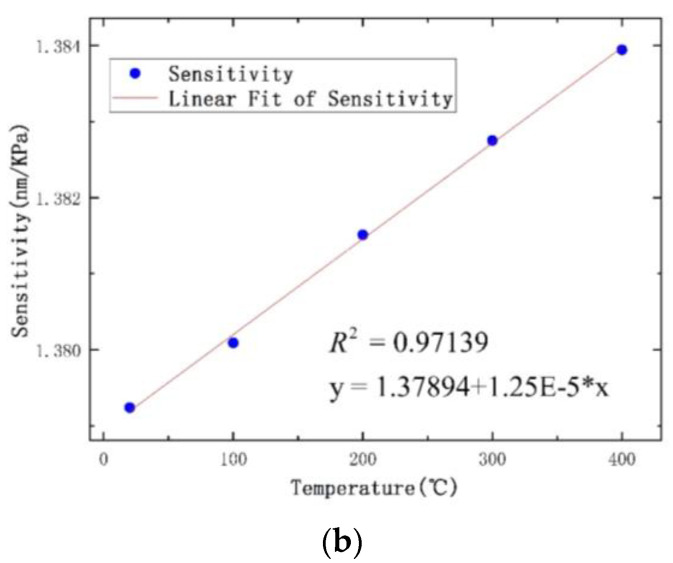
(**a**) The relationship between the length of the Fabry–Perot cavity and the temperature. (**b**) The relationship between sensitivity and temperature.

Figure 8 shows that both the zero point and the sensitivity shifted with the increase in temperature. Figure 8a shows the relationship between the length of the sensor cavity and the temperature from 20 °C to 400 °C, with an increment of 50 °C. At high temper-atures, the material deformed with thermal expansion. The glass sleeve, tubular structure, and glass wafer were composed of the same material, so the thermal expansion coefficients of the three were identical, which had little effect on the sensitive silicon diaphragm of the sealed cavity and could be ignored. Similarly, at high temperatures, the elastic modulus and thermal expansion coefficient of silicon wafers changed with temperature, i.e., they were no longer constant values, and this change was not negligible. The zero drift in the high-temperature Fabry–Perot optical fiber pressure sensor in the sealed Fabry–Perot cavity after silicon and glass bonded at a high temperature can be approximately regarded as the zero drift in the sensitive silicon diaphragm. From the simulation results, it can be concluded that the deflection of the center position of the sensitive silicon diaphragm increases with temperature, and this change can be regarded as approximately linear. Figure 8a shows that as the temperature of the Fabry–Perot cavity increased, the cavity length of the sensor decreased linearly.

The calculation showed that the temperature sensitivity of the sensor was 0.199 nm/°C. The relationship between the sensitivity and temperature of the sensor at 20, 100, 200, 300, and 400 °C is shown in Figure 8b. The structure of the sensor designed in this paper was a sealed vacuum FP cavity. The length of the FP cavity of the high-temperature pressure sensor was used to measure the pressure in the closed environment. Figure 8b shows that the sensitivity of the sensor increased with temperature.

**Figure 9:**
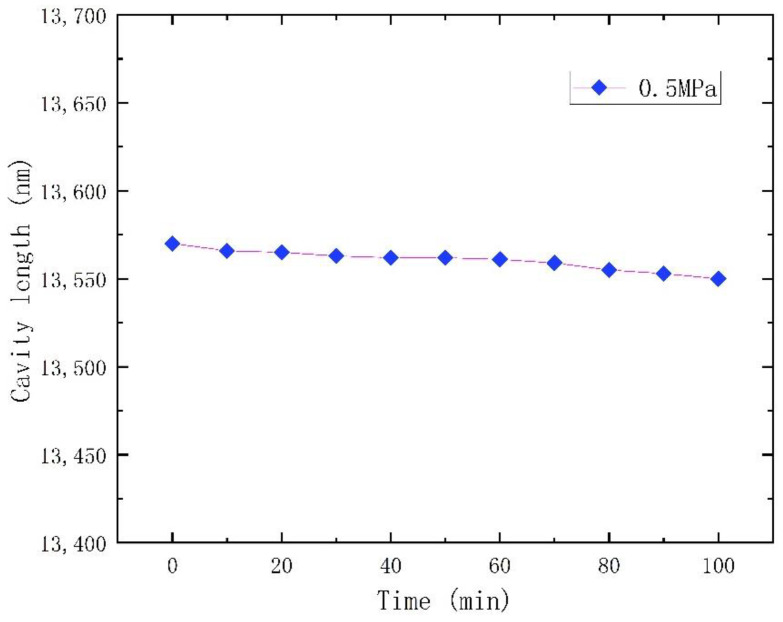
The stability over time of the proposed sensor at 0.5 MPa and 300 °C.

The stability of the sensor was measured at 300 °C for about 100 min at 0.5 MPa, as shown in Figure 9. The experimental results show that the cavity length of the sensor at 0.5 MPa decreased by 20 nm in 100 min. The slight change in the length of the sensor cavity may have been caused by the stress release of the sensor during the manufacturing process. These results indicate that the sensor remains in a stable state after releasing the stress.

## 5. Conclusions

This article introduces an optical fiber FP pressure sensor based on MEMS process and CO_2_ laser welding technology, and the sensor has been experimentally verified in a high temperature environment. Experimental results show that the maximum nonlinearity of the fiber optic FP pressure sensor is less than 1%. The pressure range is approximately 0 to 1 MPa, and the temperature range is 20 to 400 °C. The results verified the improvement of consistency and discussed the reasons for its good performance. The purpose of this design is to improve the practicability of sensitive diaphragm sensors. Combined with other designs and studies, this paper proposes that this structure can also be used for micro-fiber planar arrays. The optical fiber FP pressure sensor has the advantages of mass manufacturing, high uniformity, and low cost.

## Figures and Tables

**Table 1:** Structural parameters of the sensitive silicon diaphragm.

Performance	Symbol	Typical Value
Elastic modulus (GPa)	*E*	129.5
Poisson’s ratio	*u*	0.278
Acceleration of gravity (m/s^2^)	*g*	9.7914
Density (g/cm^3^)	*ρ*	2.33
